# Clustering Algorithm-Driven Detection of TRBC1-Restricted Clonal T-Cell Populations Produces Better Results than Manual Gating Analysis

**DOI:** 10.3390/ijms26010170

**Published:** 2024-12-28

**Authors:** Simon Buček, Andreja Brožič, Simona Miceska, Gorana Gašljević, Veronika Kloboves Prevodnik

**Affiliations:** 1 Department of Cytopathology, Institute of Oncology, Zaloška Cesta 2, 1000 Ljubljana, Slovenia; sbucek@onko-i.si (S.B.);; 2 Faculty of Medicine, University of Ljubljana, Korytkova Ulica 2, 1000 Ljubljana, Slovenia; 3 Department of Pathology, Institute of Oncology, Zaloška Cesta 2, 1000 Ljubljana, Slovenia; 4 Faculty of Medicine, University of Maribor, Taborska Ulica 8, 2000 Maribor, Slovenia

**Keywords:** flow cytometry, TRBC1, clonality, Phenograph, t-SNE, T-cell lymphoma, T-CUS

## Abstract

Flow cytometric (FC) immunophenotyping and T-cell receptor (TCR) gene rearrangement studies are essential ancillary methods for the characterisation of T-cell lymphomas. Traditional manual gating and polymerase chain reaction (PCR)-based analyses can be labour-intensive, operator-dependent, and have limitations in terms of sensitivity and specificity. The objective of our study was to investigate the efficacy of the Phenograph and t-SNE algorithms together with an antibody specific for the TCR β-chain constant region 1 (TRBC1) to identify monoclonal T-cell populations. FC- and PCR-based clonality analyses were performed on 275 samples of T-cell lymphomas, B-cell lymphomas, and reactive lymphocytic proliferations. Monotypic T-cell populations were identified in 65.1% of samples by manual gating and 72.4% by algorithm-driven analysis, while PCR-based analysis detected clonal T cells in 68.0%. Of the 262 monotypic populations identified, 46.6% were classified as T-cell lymphomas and 53.4% as T-cell populations of uncertain significance (T-CUS). Algorithm-driven gating identified monotypic populations that were overlooked by manual gating or PCR-based methods. The study highlights the difficulty in distinguishing monotypic populations as T-cell lymphoma or T-CUS. Further research is needed to establish criteria for distinguishing between these populations and to improve FC diagnostic accuracy.

## 1. Introduction

Mature T-cell lymphomas represent a large group of aggressive haematological malignancies characterised by the presence of malignant clonal T-cells, and they account for 10% to 15% of all non-Hodgkin’s lymphomas [1]. Accurate and timely diagnosis is important to achieve better outcomes for these patients. However, the flow cytometric (FC) diagnosis of T-cell lymphomas is challenging due to the immunophenotypic abnormalities inherent in these malignancies, overlap with reactive T-cells, and limitations of current T-cell clonality assays. Mature T-cell lymphomas, such as peripheral T-cell lymphoma (PTCL) not otherwise specified (NOS), nodal T-follicular helper (nTFH) cell lymphoma angioimmunoblastic-type (AITL), Sézary syndrome (SS), and other types, often exhibit a variety of clinical, morphological, immunophenotypic, and molecular features that make them difficult to recognise and classify [2,3,4]. Consequently, accurate FC immunophenotypic studies are an important part of the histopathological characterisation of T-cell lymphoproliferative disorders. However, traditional manual gating analysis is labour-intensive and potentially less accurate than novel clustering algorithm methods [5]. In addition, polymerase chain reaction (PCR)-based T-cell receptor (TCR) gene rearrangement studies, although useful for clonality identification, have limitations in sensitivity and specificity [6,7].

The TRBC1 antibody (clone JOVI-1) is an important new tool for the determination of T-cell clonality by FC. It specifically targets the T-cell receptor β-chain constant region 1 (TRBC1), distinguishing it from TCR β-chain constant region 2 (TRBC2). This specificity enables the identification of monoclonal T-cell populations based on TRBC1 expression, providing a more precise means of characterising T-cell proliferations [8]. Studies have shown that the assessment of TRBC1 expression by FC can effectively identify monotypic T-cell populations, which is crucial for the diagnosis of T-cell lymphomas [9,10]. Shi et al. showed that a single anti-TRBC1 antibody rapidly identifies clonal T-cell populations in mature CD4-positive T-cell neoplasms with a 100% sensitivity. In 16% of reactive T-cell proliferations, the authors found small CD8-positive clonal populations, which are often associated with autoimmune disorders or viral infections [9]. Novikov et al. found that this method has a sensitivity of 97% and a specificity of 91% for diagnosing peripheral T-cell lymphomas, suggesting that it could be used as a cost-effective diagnostic tool [10]. For cutaneous T-cell lymphomas (CTCL), Horna et al. showed that TRBC1 staining accurately identifies clonal Sézary cells and outperforms traditional FC and PCR methods [11]. Similarly, Shi, et al. and Muñoz-García, et al. confirmed the utility of TRBC1 in distinguishing between benign clonal T-cell expansions and T-large granular lymphocytic leukaemia (T-LGLL) in peripheral blood (PB) or bone marrow (BM), even in cases where the phenotypic aberrations are absent [12,13]. Wang et al. combined PD-1 with TRBC1 for the diagnosis and monitoring of AITL, including minimal residual disease (MRD), and showed high sensitivity and specificity [14]. Moreover, Berg, et al. and Chen, et al. identified TRBC1 as a diagnostic marker for mature T-cell lymphomas that reliably assesses clonality in different types of samples [15,16], while Waldron, et al. validated TRBC1 as an effective test with a 97% concordance rate with PCR-based clonality tests [17]. Altogether, these results suggest that TRBC1 expression analysis by FC is a valuable tool for the diagnosis of T-cell lymphomas.

On the other hand, recent advances in computational biology offer promising alternatives to traditional methods of analysis. Algorithms such as Phenograph, a clustering algorithm, and t-SNE (t-distributed stochastic neighbour embedding), a dimensionality reduction technique, enable more accurate and efficient analysis of the high-dimensional FC data. Both can analyse complex data sets to identify distinct cell populations that may be overlooked by manual gating. These algorithms have shown their potential for various applications, including the detection of rare cell populations and the identification of cellular heterogeneity in tumours [18,19].

Several studies have demonstrated the effectiveness of these algorithms in the identification of aberrant and malignant cell populations. For example, Ferrell et al. used t-SNE to identify rare and phenotypically distinct cell populations in acute myeloid leukaemia (AML) [20]. The same method can be applied to T-cell neoplasms by adapting it to focus on T-cell markers. Similarly, Levine et al. showed that Phenograph can distinguish between different subpopulations within heterogeneous samples, providing insights into disease states that could not be detected by manual gating [18]. On the other hand, DiGiuseppe et al. highlighted the combined use of Phenograph and viSNE to facilitate the identification of abnormal T-cell populations in routine clinical FC data and emphasised their potential to improve diagnostic accuracy [21]. Horna et al. demonstrated the use of semi-automated analysis to detect Sézary cells using an unsupervised clustering tool from the EuroFlow consortium. In this approach, CD4+ T cells were categorised into different clusters, and each cluster was tested for clonality based on TRBC1 expression. The results emphasise the complex immunophenotypic properties of Sézary cells and the improved detection accuracy achieved by automated methods [22].

In this study, our objective was to evaluate the effectiveness of the Phenograph and t-SNE algorithms in conjunction with an antibody specific for the β-chain constant region 1 of the T-cell receptor (TRBC1) to identify monoclonal T-cell populations. By comparing these advanced computational methods with a traditional manual gating analysis in FC- and PCR-based clonality assays, we aimed to determine whether these algorithms can provide more accurate and reliable diagnostics for mature T-cell lymphomas.

## 2. Results

Among all 275 samples analysed in our study, manual and algorithmic gating analysis with the TRBC1 antibody identified monotypic T-cell populations in 65.1% and 72.4% of samples, respectively, while a PCR-based clonality analysis showed clonal T-cells in 68.0% of samples. In 25.1% of samples, more than one monotypic T-cell population was identified. In total, 262 monotypic T-cell populations were identified, of which 46.6% were classified as T-cell lymphoma populations and 53.4% as T-cell populations of uncertain significance (T-CUS).

### 2.1. Manual and Algorithmic Gating Analysis of TRBC1 and T-Cell Lineage Antigen Expression

Manual gating analysis with the TRBC1 antibody revealed monotypic T-cell populations in 179 (65.1%) of the 275 samples and polytypic T-cells in 96 (34.9%) samples. Of the 127 mature T-cell lymphomas, 120 (94.5%) samples contained monotypic T-cell populations. In addition, 59 (39.9%) of the B-cell lymphoma and reactive lymphocytic proliferation (RLP) samples (n = 148) also had monotypic T-cell populations (Table 1).

Using the TRBC1 antibody with the Phenograph/t-SNE algorithm, monotypic T-cell populations were identified in 199 (72.4%) of the 275 samples. In the mature T-cell lymphomas (n = 127), monotypic populations were found in 122 (96.1%) samples. Only in five (3.9%) cases of mature T-cell lymphomas, no monotypic T-cell populations were found. In B-cell lymphomas and RLP (n = 148), monotypic T-cell populations were detected in 77 (52.0%) samples (Table 1).

Statistically significant differences were found between the results of manual gating and algorithmic analysis using the TRBC1 antibody to detect monotypic T-cell populations (McNemar’s test, *p* < 0.001). The algorithmic analysis detected monotypic populations in 20 (7.3%) additional samples compared to the manual analysis. Therefore, all manual analyses were reviewed and re-analysed. This showed that monotypic populations were found in all samples originally overlooked by the manual analysis, including two cases of T-cell lymphomas. Consequently, the revised results showed complete agreement between the manual and algorithmic analyses, with no statistically significant differences (McNemar’s test *p* = 1.000).

**Figure 1:**
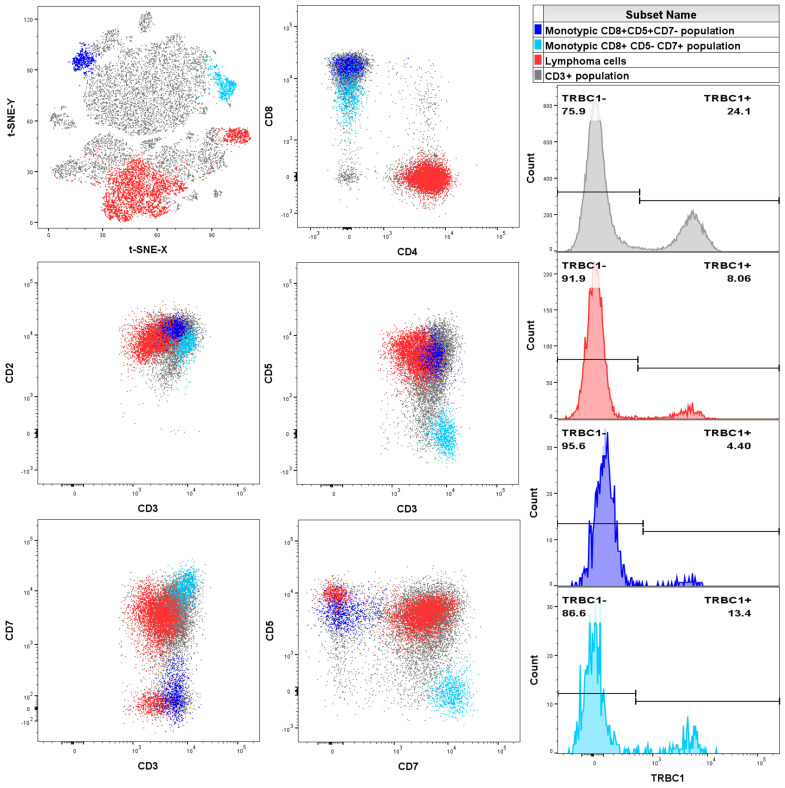
Case of a peripheral blood sample from a patient with a histopathologically confirmed diagnosis of nodal T-follicular helper cell lymphoma, angioimmunoblastic type. Initial manual analysis of the CD3-positive population (grey) overlooked the monotypic CD4-positive lymphoma population (red) and the CD8-positive/CD7-negative T-cell clones of uncertain significance (T-CUS) (dark blue) due to overlap with polyclonal T cells. It only showed the presence of monotypic CD8-positive/CD5-negative T-CUS (light blue). However, Phenograph/t-SNE analysis identified overlooked monotypic CD8-positive T-CUS populations, as well as a CD4-positive population with a monotypic expression pattern of TRBC1. A revised manual analysis confirmed the presence of all three monotypic populations and the entire monotypic CD4 population was classified as a lymphoma population based on the expression of CD279 and CD10 (data from the FC diagnostic T-cell lymphoma antibody panel).

Figure 1 shows FC data from a PB sample of a patient diagnosed with T-cell lymphoma. Monotypic populations, including the T-cell lymphoma population, were overlooked by manual analysis but were successfully identified by Phenograph and t-SNE analysis. The full initial and revised manual flow cytometry analyses can be viewed in Supplementary Material S1.

**Figure 2:**
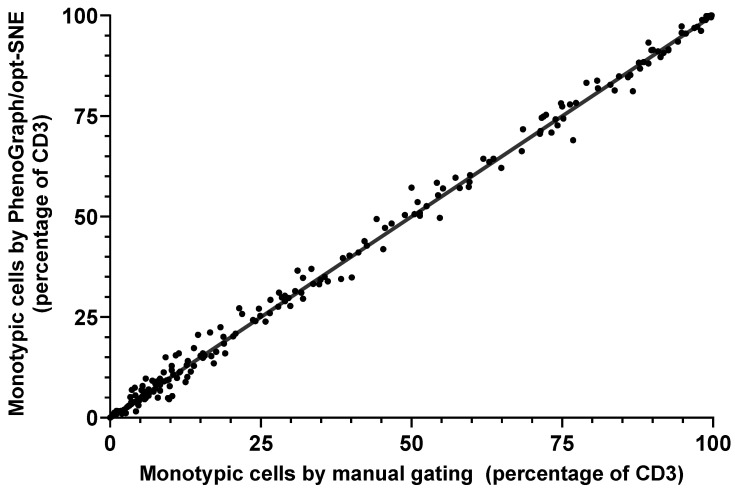
Correlation between manual gating and PhenoGraph/t-SNE analysis for monotypic cell population percentages (rs = 0.9919, *p* < 0.0001).

On the other hand, no statistically significant differences were found between the size of the monotypic T-cell populations determined by manual and algorithmic analyses (Wilcoxon signed-rank test, *p* > 0.05). The correlation of T-cell population sizes from both analyses showed strong statistical significance (Spearman’s correlation, rs = 0.9919, *p* < 0.0001) (Figure 2).

Our analysis showed that the monoclonal T-cell populations overlooked by manual gating were very small. The average size of the overlooked populations was 0.25% of CD45-positive cells, ranging from 0.031% to 0.54%.

Similarly, no significant differences were observed between the two analyses’ results for the median fluorescence intensities (MFI) values for CD2, CD3, CD4, CD5, CD7, CD8, and CD45 antigen expression (Wilcoxon signed-rank test *p* > 0.05). The correlation between the results of the two methods was also very high (Spearman’s correlation, rs > 0.98) and statistically significant (*p* < 0.0001). 

### 2.2. Results of the PCR-Based Analysis of T-Cell Clonality

A PCR analysis confirmed the presence of a T-cell clonal population in 187 (68.0%) of the 275 samples included in the study (Table 1), with 150 (80.2%) samples classified as clonal and 37 (19.8%) detected as clonal with a polyclonal background. Polyclonal T-cells were identified in 88 (32.0%) of the samples. In the mature T-cell lymphomas, PCR detected clonality in 119 (93.7%) of the 127 samples, with 110 (92.4%) samples classified as clonal and 9 (7.6%) as clonal with a polyclonal background. In the mature B-cell lymphoma and RLP samples (n = 148), PCR confirmed the clonality in 69 (46.6%) samples, with 40 (58.0%) samples classified as clonal and 29 (42.0%) showing clonality with a polyclonal background.

When comparing the PCR results with the algorithmic FC method, a statistically significant difference was found (*p* = 0.019, McNemar’s test). Discrepancies between the PCR and the FC analyses were found in a minority of samples. In particular, in four (1.5%) samples where a clonal population was detected by PCR, TRBC1-based FC (both manual and algorithmic) could not confirm this finding (Table 2). These discrepant cases involved three patients with B-cell lymphoma (two cases of diffuse large B-cell lymphoma (DLBCL) in the lymph nodes and one case of chronic lymphocytic leukaemia/small lymphocytic lymphoma (CLL/SLL) in BM) and one patient with RLP in a lymph node sample. Despite re-analysis with the adjusted Phenograph parameters (κ coefficient of 20) and modified t-SNE settings, these cases remained unresolved.

In contrast, by using TRBC1, monotypic populations were identified in 16 (5.8%) samples where PCR failed to detect clonality. These missed cases included 3 mature T-cell lymphomas, 10 mature B-cell lymphomas and 3 cases of RLP in 10 BM samples, 4 lymph node biopsies, and 2 PB samples (Table 2). In most cases, the monotypic population was smaller than 1%. The average size of these monotypic populations was 0.3% of CD45-positive cells and ranged from 0.01% to 2.34%. One exception was a BM sample, where the monotypic population accounted for 2.34% of the CD45-positive cells.

### 2.3. Detailed Analysis of the Monotypic T-Cell Populations

In total, 262 monotypic T-cell populations were identified in all lymphoma and RLP samples when using the Phenograph/t-SNE algorithm. Of these populations, 122 (46.6%) were classified as T-cell lymphoma populations and 140 (53.4%) as T-CUS.

**Figure 3:**
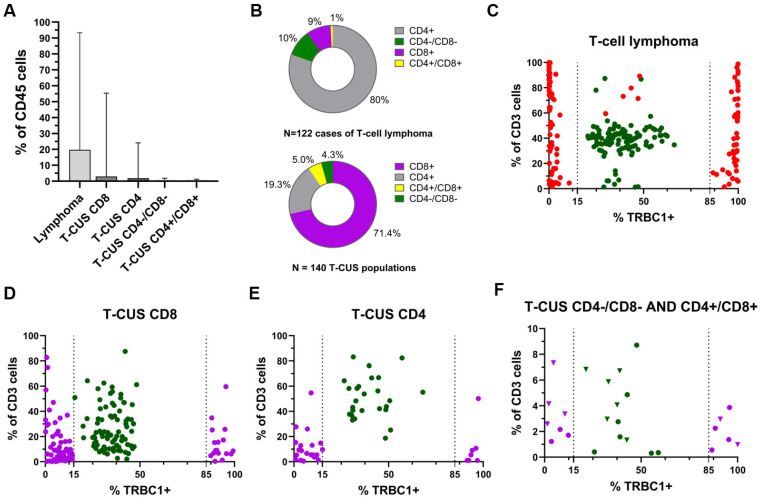
Comparison of monotypic T-cell lymphoma populations and T-cell populations of uncertain significance (T-CUS) in cases with mature T-cell and B-cell lymphomas and with reactive lymphocytic proliferations (RLP). (**A**) The size comparison of monotypic T-cell lymphoma and T-CUS populations. (**B**) Incidence and basic phenotypic features of T-cell lymphomas (top) and T-CUS (bottom). (**C**) T-cell lymphoma cases with monotypic lymphoma populations (red dots) and polytypic background T-cells (green dots). (**D**–**F**) Cases with T-CUS populations: CD4+, CD8+, double positive (purple dots) and double negative (purple triangles). Polytypic background T-cells are shown in green dots and triangles. The dotted lines in diagrams (**C**–**F**) represent the boundaries between monotypic T-cell populations and polytypic background T-cells (if the cells expressed less than 15% or more than 85% of TRBC1, they were considered monoclonal.

Of the T-cell lymphoma populations, 98 (80%) were CD4-positive, 12 (10%) were CD4/CD8-double-negative, 11 (9%) were CD8-positive and 1 (1%) was CD4/CD8-doublepositive (Figure 3B,C). The average size of these lymphoma populations was 19.68% of the CD45-positive cells, with a range from 0.033% to 93.37% (Figure 3A). The smallest lymphoma population detected in a PB sample from a patient with SS contained 348 events.

In contrast, CD8-positive populations predominated in T-CUS. They were observed in 100 (71.4%) cases, while 27 (19.3%) were CD4-positive, 7 (5.0%) CD4/CD8-double-positive and 6 (4.3%) CD4/CD8-double-negative (Figure 3B,D–F). The average size of the T-CUS populations was 2.50% of CD45-positive cells and ranged from 0.014% to 55.40% (Figure 3A). The smallest T-CUS population detected in a PB sample from a patient with CLL/SLL was CD8-positive and consisted of 82 events.

Among the 199 samples with monotypic populations, multiple different monotypic T-cell populations were observed in 50 (25.1%) samples, representing either T-cell lymphomas or T-CUS populations. Two different monotypic populations were observed in 38 (19.1%) samples, three in 11 (5.5%) samples, and four (0.5%) in one sample.

In the 127 samples diagnosed with mature T-cell lymphomas, a lymphoma population was detected in 91 (71.7%) cases. However, an additional T-CUS population was found in 21 (16.5%) of these samples, with two T-CUS populations detected in 9 (7.1%) cases and 3 T-CUS populations detected in 1 case (0.8%).

The majority of T-CUS populations in patients with mature T-cell lymphomas were observed in PB and BM. Nevertheless, in nine (7.1%) cases, a single T-CUS population was detected in samples from lymph nodes. In all cases, the T-CUS populations were CD8-positive. T-CUS was found in four AITLs, four PTCLs, NOS, and one SS.

Among the 148 samples from patients with B-cell lymphoma or RLP, T-CUS populations were detected in 54 (36.5%) samples from patients with B-cell lymphoma (19 DLBCL, 13 follicular lymphomas (FL), 13 marginal zone lymphomas (MZL), 4 mantle cell lymphomas (MCL), 3 classic Hodgkin lymphomas (cHL), and 2 CLL/SLL. In addition, T-CUS populations were detected in 23 (15.5%) cases with RLP.

The majority of the T-CUS populations in patients with B-cell lymphoma and RLP were observed in the PB and BM samples. Of the 74 BM and PB samples, one T-CUS population was found in 46 (62.2%) samples, two T-CUS populations in 17 (23.0%) and three T-CUS populations in 2 (2.7%) cases.

In the lymph node biopsy samples (n = 74), a T-CUS population was identified in 11 (14.9%) cases of B-cell lymphoma (4 DLBCL, 3 MZL, 2 FL, 2 MCL, and 1 CLL/SLL). Ten (83.3%) cases of T-CUS populations were CD8-positive and one case (8.3%) was CD4-negative/CD8-negative. A CD8-positive T-CUS population was also identified in one case (8.3%) of RLP, although this sample was contaminated with PB.

## 3. Discussion

This study presents an in-depth comparison of manual gating, PCR-based clonality assays, and advanced computational techniques (Phenograph and t-SNE) for detecting monotypic and clonal populations in patients with T-cell and B-cell lymphomas, cHL, and RLP. Our findings highlight the advantages and limitations of each method, offering critical insights into the practical applications of these diagnostic tools in clinical practice.

### 3.1. Comparison of Phenograph/t-SNE and Manual Gating

Monotypic populations were detected by Phenograph and t-SNE in 199 (72.4%) of the 275 samples, outperforming manual gating, which identified these populations in only 179 (65.1%) of the samples. The increased sensitivity of the Phenograph when analysing multiple parameters facilitated the precise identification of small monotypic populations, as demonstrated by its application to detect rare AML phenotypes [18] or to identify aberrant T-cell populations [21]. The capabilities of unsupervised clustering algorithms, such as Phenograph and t-SNE, help uncover complex cellular patterns that could be obscured by user bias when analysed manually [23].

The manual analysis failed to detect 20 monotypic populations identified by the algorithmic approach, including three cases of T-cell lymphoma. The limited sensitivity of manual analysis in detecting certain monotypic populations can be attributed to two main factors: the small sizes of these populations and the minimal differences in antigen expression. The average size of monotypic populations overlooked by manual gating was 0.25% of CD45-positive cells, making detection challenging when using conventional visual gating methods that can miss small populations [21]. In addition, minimal differences in antigen expression between monotypic populations, such as lymphomas or T-CUS and reactive polyclonal T-lymphocytes, could affect manual analysis and cause monotypic populations to be overlooked [17]. In contrast, algorithmic methods, such as Phenograph and t-SNE, which compare all markers simultaneously, provide a more successful detection of these subtle differences that may be overlooked by traditional manual analysis [5].

Advanced computer algorithms, such as Phenograph and t-SNE, were therefore critical in our study for identifying the subtle changes in the expression of T-cell antigens in T-cell lymphoma and T-CUS populations that may be overlooked by manual gating in FC. These algorithms are excellent at recognising rare populations and clustering biologically meaningful subgroups. However, as Pedersen and Olsen emphasise, these methods do not provide a completely unsupervised solution and require iterative interpretation by subject matter experts to achieve accurate and clinically meaningful results, which emphasises the need to integrate computational tools with expert analysis for accurate diagnosis [23].

### 3.2. Discrepancies Between Algorithmic and PCR Results

The algorithmic approach to FC analysis is highly sensitive and capable of detecting subtle differences in marker expression, which allows for the identification of distinct cellular populations [18]. However, this sensitivity depends on the measurable variability of the antigen expression. If the antigens used (such as CD2, CD3, CD4, CD5, CD7, and CD8) do not show altered expression, the method might fail to detect a monotypic or clonal population [21]. This limitation may explain the discrepancy as to why, in 1.5% of cases where PCR confirmed the presence of a clonal population, FC using a TRBC1-based method did not confirm these findings.

One possible solution would be to expand the panel of T-cell markers in FC with antibodies against CD26, CD279 (PD-1), CD10, CD30, CD56, and CD57, which would improve the detection of clonal T-cells and increase the diagnostic accuracy in various lymphomas. Antigens such as CD279 and CD10 are particularly effective in the detection of AITL, as CD279 is often overexpressed in AITL. The use of CD279 in combination with CD10 improves the ability to distinguish AITL from RLPs or T-CUS [2,3,14]. Similarly, CD26 helps in the identification of SS when used in combination with CD4 and CD7 [11,22]. Markers such as CD56 and CD57 are valuable for the identification of clonal T-cell populations in T-LGLL [12,13] and other T-cell lymphomas with cytotoxic properties [4,10].

Furthermore, the use of a TRBC1-based FC in our study proved to be highly effective for the detection of monotypic T-cell populations, especially small populations that might be missed by molecular methods such as PCR. The recent inclusion of a TRBC2 antibody in diagnostic panels represents a significant advance. Dual TRBC1/TRBC2 staining, as demonstrated by Horna et al., mitigates the artefacts associated with single antibody analysis, thereby increasing the reliability of clonality assessment. This approach simplifies the distinction between polytypic and monotypic T-cell populations and significantly improves diagnostic accuracy in unclear cases [24]. Unfortunately, the TRBC2 antibody was not yet available at the time of the start of this study. However, we plan to include this antibody together with TRBC1 in our future T-cell lymphoma FC panels. To further refine the diagnostic methods, it is important to continue to evaluate which antibodies contribute most effectively to these FC panels.

In 16 samples identified by the Phenograph/t-SNE algorithm, PCR could not detect clonal populations. One of the reasons for this discrepancy could be the size of the detected populations. In all but one case, the size of the populations missed by PCR was less than 1% of the CD45-positive cells. The BIOMED-2 PCR method can only reliably detect clonal populations if they account for at least 1% of the cells in the sample [25]. This limitation indicates that smaller clonal populations may remain undetected [7,25,26]. In one particular case of bone marrow, in which the clonal population accounted for more than 2% of the CD45-positive cells, PCR was still unable to detect it. The likely explanation for this phenomenon is that the non-lymphoid cells present in the sample that are not detected in the CD45 gate, such as erythroblasts, diluted the proportion of clonal cells so that the total proportion fell below the detection limit of the PCR. Other factors could also contribute to the limitations of PCR. The BIOMED-2 method relies on specific gene rearrangements to identify clonal populations. Rare or unusual rearrangements may be missed if they are not targeted by the standard primer sets used, reducing the accuracy of the test. Technical errors, such as inefficient DNA extraction or degradation, can also affect the efficacy of PCR, but in all overlooked cases, DNA quality control showed DNA of sufficient quality for the assay [7,27]. These challenges highlight the importance of using complementary methods such as FC to detect small monotypic clonal populations at the early stages of disease or to monitor MRD [14,27].

### 3.3. Failure to Detect Monotypic Populations in T-Cell Lymphoma Cases

Our study also identified five cases of T-cell lymphomas (two angioimmunoblastic T-cell lymphomas, two cases of nodal T-follicular helper cell lymphoma, NOS, and one peripheral T-cell lymphoma, NOS) in which neither FC (manual or algorithmic analysis) nor PCR could confirm the presence of a monotypic population.

Three of these patients were being treated with corticosteroids for previous rheumatological diseases at the time of diagnosis, which may have influenced the diagnostic process. Corticosteroids can cause immunosuppression, reduce the number of detectable lymphoma cells in the sample, and consequently, make it more difficult to identify a monotypic population, regardless of the method used [28].

In one case, the biopsy showed the presence of necrosis, which likely interfered with the diagnostic process. Necrosis leads to the destruction of lymphoma cells and limits the ability of FC and PCR to recognise clonal populations. Dead and degenerated cells that form necrotic tissue are usually excluded from FC analysis [21], and in PCR, the presence of necrosis could interfere with the results due to DNA degradation [17,27].

However, no clear pre-analytical explanation could be found for the fifth case. The sample was of good quality and no previous therapies were reported that could have influenced the analysis. We suspect that this case may be a rare T-cell lymphoma with no detectable clonal population. In about 5 to 10 per cent of T-cell lymphoma cases, PCR cannot detect the presence of a clonal population [7,26]. The inclusion of additional markers in the FC panel could potentially help to resolve negative results and identify aberrant populations, but no marker for T-cell antigens included in the TRBC1 tube or the expanded diagnostic T-cell panel showed aberrant expression.

The lack of detectable clonal populations with PCR and FC could also explain the previously mentioned cases.

### 3.4. Influence of Monotypic T-CUS Populations on the Diagnosis of B-Cell and T-Cell Lymphomas

The detection of clonal T-cell populations in clinical laboratory practice is usually interpreted as a potential sign of malignant T-cell lymphoma. However, clonal T-cell proliferation can also occur in response to chronic viral infections, such as the cytomegalovirus [29] and the Epstein–Barr virus [30], as well as completed acute infections [31], autoimmune diseases, neoplastic processes, or other antigen exposures [32]. This makes it difficult to distinguish between malignant T-cell clones and reactive or benign clonal expansions, such as T-CUS.

We identified T-CUS populations in 52% of cases without diagnostic evidence of T-cell lymphoma. Of these, 36.5% were observed in cases diagnosed with mature B-cell lymphoma and 15.5% were found in cases of RLP. In addition, T-CUS was detected in 24.4% of cases with mature T-cell lymphomas. These results are consistent with the literature reporting the occurrence of clonal T-cell populations in both malignant and reactive settings [12,33]. However, we observed a higher prevalence of T-CUS in B-cell lymphomas and RLP compared to published data. For example, Shi et al. reported T-CUS populations in 26% of cases in patients and 13% in healthy adult donors [12]. The higher prevalence of T-CUS in our study may be due to the significantly larger number of patients diagnosed with different types of lymphoma at the different stages of treatment or after the completion of lymphoma treatment.

Most mature T-cell lymphomas in our cohort were CD4-positive and were characterised by abnormal immunophenotypic features, including an altered expression of antigens of the T-cell lineage (e.g., CD2, CD3, CD5, and CD7), and abnormal expression of additional antigens such as CD279 and CD10. These features distinguish lymphoma T-cell clones from T-CUS, which are predominantly derived from CD8 and usually show only minor deviations from the normal T-cell immunophenotypes [11,14]. This contrast supports the use of comprehensive immunophenotyping to differentiate between malignant and benign populations. The diagnostic challenge arises in cases of CD8-positive mature T-cell lymphomas, as these may immunophenotypically overlap with CD8-positive T-CUS. However, the presence of aberrations in the expression of T-cell markers together with clinical and molecular findings usually enables reliable differentiation [12]. The immunophenotypic characteristics of the CD8-positive T-CUS found in our study were consistent with the previously published literature [9,12,33].

Similarly, CD4-positive T-CUS, although rarely observed, may pose a diagnostic challenge due to their similarity to certain T-cell lymphoma types. In our study, we identified CD4-positive T-CUS in 9.8% of all cases, representing 19.3% of all T-CUS populations. However, the published information on CD4-positive T-CUS populations is limited. We found only two studies describing CD4-positive T-CUS. One reported its occurrence in 1% of all samples [12] and the other in 10.4% of all samples, but this number included CD4-positive and CD4/CD8-double-positive T-CUS populations with a strong or weak expression of CD4 [33].

The most prominent immunophenotypic feature in all CD4-positive T-CUS cases was the loss or decreased expression of CD7 compared to normal CD4-positive polyclonal cells, in addition to the variable expression of other T-cell antigens. These findings are consistent with those of Pu et al. [33]. However, given the limited number of CD4-positive T-CUS populations detected, more detailed analyses are required to accurately determine the immunophenotypic characteristics of CD4-positive T-CUS populations and to understand their impact on the diagnosis of mature CD4-positive T-cell lymphomas, particularly in those types where the loss of CD7 expression is common.

In B-cell lymphomas, the presence of T-CUS likely represents an immune response to lymphoma cells in the tumour microenvironment or to opportunistic infections that are common in immunocompromised patients [12,24,33]. While T-CUS in B-cell lymphomas generally does not pose diagnostic challenges, rare cases of composite B-cell and T-cell lymphomas may require detailed immunophenotypic and molecular investigations to differentiate between coexisting malignancies. Similarly, T-CUS in RLPs can complicate the diagnosis, especially in fine-needle aspiration biopsy (FNAB) samples used to differentiate reactive lymph nodes from lymphoma. In such cases, clinical features, cytomorphology, and molecular analyses are crucial. When necessary, follow-up biopsies and advanced techniques are also used to ensure accurate diagnosis and appropriate patient management [34].

## 4. Materials and Methods

### 4.1. Patients and Sample Selection

A total of 275 samples from 221 patients referred to the Institute of Oncology in Ljubljana for FC analysis were included in the study. The samples were of different origin, as shown in Table 3. Of these, 127 samples originated from patients diagnosed with mature T-cell lymphomas, while the remaining 148 samples were from patients with mature B-cell lymphomas or RLP (Table 4).

To distinguish patients with mature T-cell lymphomas from those with no clinical evidence of current or previous T-cell malignancy, medical histories were reviewed. This review included pathology reports, laboratory test results (including FC reports), clinical notes, and imaging reports. The study was approved by the National Medical Ethics Committee of the Republic of Slovenia (approval number 0120-165/2020/4) and was performed in compliance with the Helsinki Declaration.

### 4.2. Flow Cytometry

The preparation of the samples for FC varied depending on the sample type. PB and BM aspirate samples were collected in EDTA tubes, while tissue samples (lymph nodes, bone marrow, or skin biopsies) and FNAB were collected in tubes containing 1 mL in house cell collection medium (4.5% bovine serum albumin, 0.45% EDTA in a phosphate buffer solution containing 90,000 IU/mL penicillin and 4 µg/mL garamycin). Tissue samples were further disintegrated using the gentleMACS™ Dissociator and C Tubes (Miltenyi Biotec, Bergisch Gladbach, Germany) to prepare single-cell suspensions in a 1 mL cell collection medium. The body fluids were centrifuged at 1500 rpm (Hettich, Universal 32 centrifuge, Tuttlingen, Germany) and also resuspended in 1 mL of cell collection medium. The CSF samples were transported on ice within half an hour of collection.

All samples were filtered through a 50 μm pore filter (CellTrics; Sysmex Corporation, Kobe, Japan) and the cells were counted using a Sysmex XP-300 haematology analyser (Sysmex Corporation, Kobe, Japan) For each sample, 500,000 cells were used, except in the case of paucicellular samples, where the entire sample was processed. The number of required cells was transferred to a new 5 mL polystyrene tube (Falcon Corning, Corning, CA, USA), washed with 4 mL PBS buffer saline, and centrifuged at 1500 rpm for 5 min. The supernatant was discarded, and the remaining cells were incubated with ten monoclonal antibodies (CD2, CD3, CD4, CD5, CD8, CD19, CD45, TCRγδ (all from BD Biosciences, San Jose, CA, USA), CD7 (Miltenyi Biotec, Bergisch Gladbach, Germany), and anti-TRBC1, clone JOVI-1 (Ancell Corporation, Stillwater, MN, USA) for 20 min in the dark at room temperature. The erythrocytes from the PB and BM samples were then lysed with BD FACS Lysing Solution (BD Biosciences, San Jose, CA, USA) for 10 min at room temperature. The FC analysis was performed within 48 h of sample collection for all the samples.

A 10-colour FACSCanto flow cytometer (BD Biosciences, San Jose, CA, USA) was used to detect at least 100,000 CD45-positive events for the BM and PB samples and 50,000 CD45-positive events for all other samples. For the paucicellular samples, all CD45-positive events were detected. The FC measurements and manual analyses were performed using BD FACSDiva software version 9.1 (BD Biosciences, San Jose, CA, USA). The Phenograph and t-SNE analyses were performed using FlowJo software version 10.9.0 (Becton, Dickinson and Company, Franklin Lakes, NJ, USA).

T-cell populations expressing less than 15% or more than 85% TRBC1 were considered clonal [9,10]. T-cell lymphoma populations were defined based on the histopathological reports and the results of the routine FC T-cell lymphoma panel used in our laboratory. Populations with immunophenotypic features consistent with mature T-cell lymphomas were considered lymphoma populations. The populations that did not fulfil these criteria were defined as T-CUS. Manual and algorithm-driven analyses were performed independently, and the results were reviewed and correlated with a PCR analysis of the T-cell receptor gene rearrangements BIOMED-2 assay (Invivoscribe, San Diego, CA, USA). Discrepant cases were reviewed by two experienced FC analysts, and consensus was reached.

#### 4.2.1. Conventional Gating Analysis

Viable cells were gated using a standard area forward scatter plot (FSC-A) versus an area side scatter plot (SSC-A) to exclude debris and dead cells. Doublets and aggregates were excluded using an FSC-A (area) versus FSC-H (height) dot plot. Major lymphocyte populations were identified sequentially based on antigen expression: T-cells (CD3-positive), B-cells (CD19-positive), and NK-cells (CD3-negative, CD7-positive). TRBC1 expression was only analysed in CD3-positive TCRγδ-negative T-cell populations. CD4-positive, CD8-positive, CD4/CD8-double-positive, and CD4/CD8-double-negative T-cells were visualised in N × N dot plots to assess the expression of CD2, CD3, CD4, CD5, CD7, CD8, and CD45 antigens. TRBC1 expression was assessed for different T-cell subsets with at least 50 events using logical scale histograms. The percentage of TRBC1-positive cells was determined, with the threshold for positivity set visually using TRBC1-negative and TRBC1-positive benign T-cell populations as controls. Clonal population size was measured as a percentage of CD3-positive cells, and the MFI was recorded for antigens CD2, CD3, CD4, CD5, CD7, CD8, and CD45.

#### 4.2.2. Phenograph and t-SNE Analysis

The Phenograph clustering algorithm was applied to the CD3-positive cells in each sample using κ-nearest neighbours ≤ 40. The gating strategy for CD3-positive cells followed the same protocol as the manual analysis. FSC-A, SSC-A, and the antigens CD2, CD3, CD4, CD5, CD7, CD8, CD45, and TCRγδ were included in both analyses. All Phenograph clusters, except TCRγδ-positive clusters, were analysed for TRBC1 expression as in the conventional analysis. To visualize the Phenograph clusters, the opt-SNE dimensionality reduction algorithm reduced the 10-dimensional data to two dimensions. The Exact (Vantage Point Tree) and Barness-Hut Gradient algorithms were used with standard parameters (iterations: 1000; perplexity: 30; and learning rate: as defined by the algorithm). The relationships between the Phenograph clusters were examined using the opt-SNE results, relevant T-cell antigen expression, and TRBC1 expression. Due to the heterogeneity of T-cell antigen expression and large population sizes, Phenograph analysis with a κ-value of 40 or less may result in over clustering. Multiple Phenograph clusters may represent a larger lymphoma or monotypic population. In such cases, the clusters were merged with Boolean gating and analysed as a single population. The size of the clonal population was determined, and the MFIs were recorded as in the conventional gating analysis.

### 4.3. T-Cell Receptor Gene Rearrangement Studies

Gene rearrangement studies were performed on all samples using the BIOMED-2 assay (Invivoscribe, San Diego, CA, USA) according to the previously published instructions [7]. The total cellular DNA was extracted, and PCR amplification was performed in five multiplex PCR tubes. ASR Biomed-2 primers (Invivoscribe, San Diego, CA, USA) were used that targeted specific regions, such as TCR Vβ, Dβ, Jβ, Vγ, and Jγ. PCR products were separated and detected by capillary gel electrophoresis on an ABI 3500 gene analyser (Applied Biosystems, Waltham, MA, USA) and analysed with fragment analysis software version 3.1 (GeneScan; Applied Biosystems, Waltham, MA, USA).

### 4.4. Statistical Analysis

Descriptive statistics were calculated to summarise the data. Mean percentages and MFI values were compared using the Wilcoxon signed-rank test for paired samples. Spearman’s rank correlation coefficient was used to assess the relationship between the results of the two FC analysis methods. The nominal data were analysed using the McNemar’s test to assess the differences between the revised FC results and the PCR results. A two-tailed *p*-value of ≤0.05 was considered statistically significant. All statistical analyses were performed using GraphPad Prism version 10.2.3 (GraphPad Software, Boston, MA, USA).

## 5. Conclusions

The diagnosis of T-cell malignancies in the presence of T-CUS populations by FC itself is challenging due to the overlapping immunophenotypic characteristics of reactive and malignant populations. In cases where the biopsy material is insufficient for whatever reason, our results highlight the need for a multi-faceted approach that integrates computational analyses, immunophenotypic panels, and molecular studies.

In particular, computational methods such as the Phenograph and t-SNE algorithms have proven to be highly effective. These methods utilise high-dimensional data analysis to detect small populations and subtle changes in antigen expression and uncover monotypic populations that are often overlooked by manual gating and not detected by PCR due to their small size. In contrast to conventional methods that rely on predetermined thresholds for classification, these algorithms adaptively identify rare and complex cell populations by revealing patterns in the data. Their ability to recognise heterogeneous populations, including those below the sensitivity threshold of PCR, underlines their significant value in supporting the diagnosis of T-cell lymphoma.

Future research and repeated testing of samples with identified T-CUS populations in the following months and years are needed to explore the biological significance of T-CUS populations, including how to improve differentiation between malignant T-cell populations and T-CUS. Such tests may provide deeper insights into the progression and development of both T-cell and B-cell lymphomas and enrich our understanding and management of these diseases. By refining diagnostic strategies and integrating advanced technologies, including computational tools such as Phenograph and t-SNE, we can greatly improve the accuracy of T-cell lymphoma diagnosis, ultimately leading to better clinical outcomes.

## Figures and Tables

**Table 1:** Comparison of results from Phenograph/t-SNE, manual rating, and PCR methods.

Method	Monotypic/Clonal Populations	Polytypic/Polyclonal Populations
Manual Gating	179 (65.1%)	96 (34.9%)
Phenograph/t-SNE	199 (72.4%)	76 (27.6%)
PCR	187 (68.0%)	88 (32.0%)

Abbreviations: PCR, polymerase chain reaction.

**Table 2:** Discrepancies in clonality detection between PCR and TRBC1-based FC in lymphoma and reactive lymphocytic proliferation samples.

Cases with Clonal/Monotypic T-Cells not Recognised by FC	4
Diffuse large B-cell lymphoma, NOS	2
Chronic lymphocytic leukaemia/small lymphocytic lymphoma	1
Reactive lymphocytic proliferation	1
**Cases with Clonal/Monotypic Cells not Recognised by the PCR Method**	**16**
Peripheral T-cell lymphoma, NOS	2
Nodal TFH cell lymphoma Angioimmunoblastic-type	1
Diffuse large B-cell lymphoma, NOS	5
Follicular lymphoma	2
Chronic lymphocytic leukaemia/small lymphocytic lymphoma	1
Marginal zone lymphoma	1
Reactive lymphocytic proliferation	4

Abbreviations: FC, flow cytometry; NOS, not otherwise specified; PCR, polymerase chain reaction; TFH, T-follicular helper.

**Table 3:** Description of the sample types included in the study.

Sample Type	Number of Samples
Lymph node biopsies ^1^	133
Bone marrows	70
Peripheral blood samples	45
Skin biopsies	13
Pleural effusions	11
Cerebrospinal fluids	2
Vitreous fluid	1
**All samples**	**275**

^1^ Lymph node biopsies contained 90 fine-needle aspiration biopsy samples and 43 tissue biopsy samples.

**Table 4:** Detailed information on the pathohistological diagnoses of patients included in the study.

Diagnosis	Number of Samples
**Mature T-cell lymphomas**	**127**
Peripheral T-cell lymphoma, NOS	53
Nodal TFH cell lymphoma Angioimmunoblastic type	29
Sézary syndrome	15
Nodal TFH cell lymphoma, NOS	12
Mycosis fungoides	9
Anaplastic large cell lymphoma ALK positive or ALK negative	4
Monomorphic epitheliotropic intestinal T-cell lymphoma	2
T-cell lymphoma, NOS ^1^	2
Primary cutaneous peripheral T-cell lymphoma, NOS	1
**Mature B-cell lymphomas**	**73**
Diffuse large B-cell lymphoma, NOS	25
Marginal zone lymphoma	18
Follicular lymphoma	16
Mantle cell lymphoma	9
Chronic lymphocytic leukaemia/small lymphocytic lymphoma	5
**Classical Hodgkin lymphoma**	**17**
**Reactive lymphocytic proliferation**	**58**

Abbreviations: NOS, not otherwise specified; TFH, T-follicular helper; ALK, anaplastic lymphoma kinase. ^1^ In one case the histopathological diagnosis was made on the basis of an examination of the bone marrow and in the other case on the basis of lung tissue. A more precise classification of T-cell lymphoma was not possible and additional diagnostic tests were not performed due to the small amount of biopsied tissue and the death of both patients.

## Data Availability

The original contributions presented in the study are included in the article; further inquiries can be directed to the corresponding author.

## Supplementary Materials

The supporting information can be downloaded at https://www.mdpi.com/article/10.3390/ijms26010170/s1.
