# Supplementary material for: Clustering Algorithm-Driven Detection of TRBC1-Restricted Clonal T-Cell Populations Produces Better Results than Manual Gating Analysis
Source: Int J Mol Sci. 2024 Dec 28;26(1):170. doi: 10.3390/ijms26010170 (PMC11720138; doi:10.3390/ijms26010170)
Supplement: Supplementary file 1 [file ijms-26-00170-s001.zip › ijms-3372768-supplementary.pdf]

Initial manual analysis where only one CD8+/CD7+/CD5- T-CUS population (light blue) was found. (page 1/4)

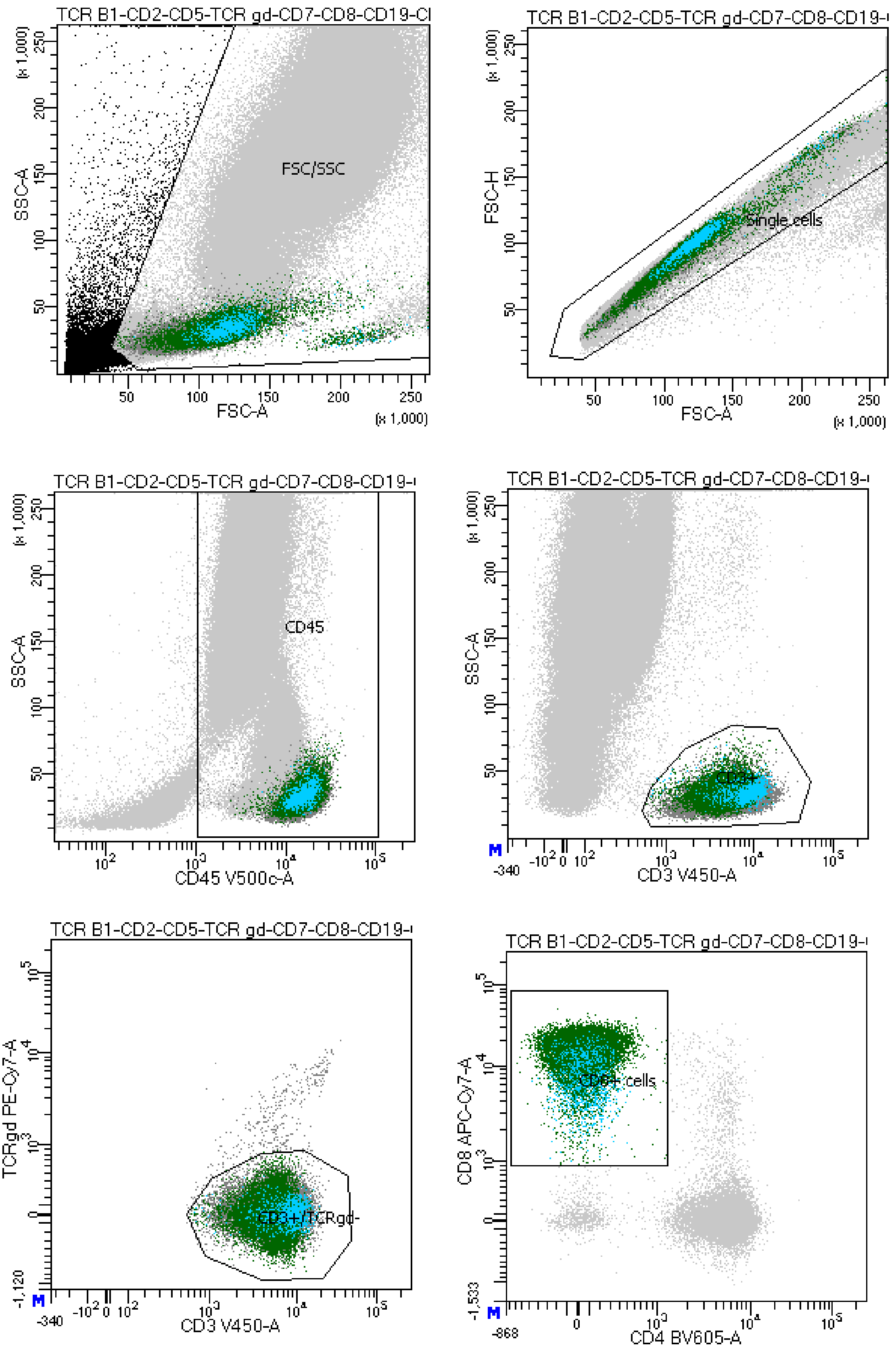

Initial manual analysis where only one CD8+/CD7+/CD5- T-CUS population (light blue) was found. (page 2/4)

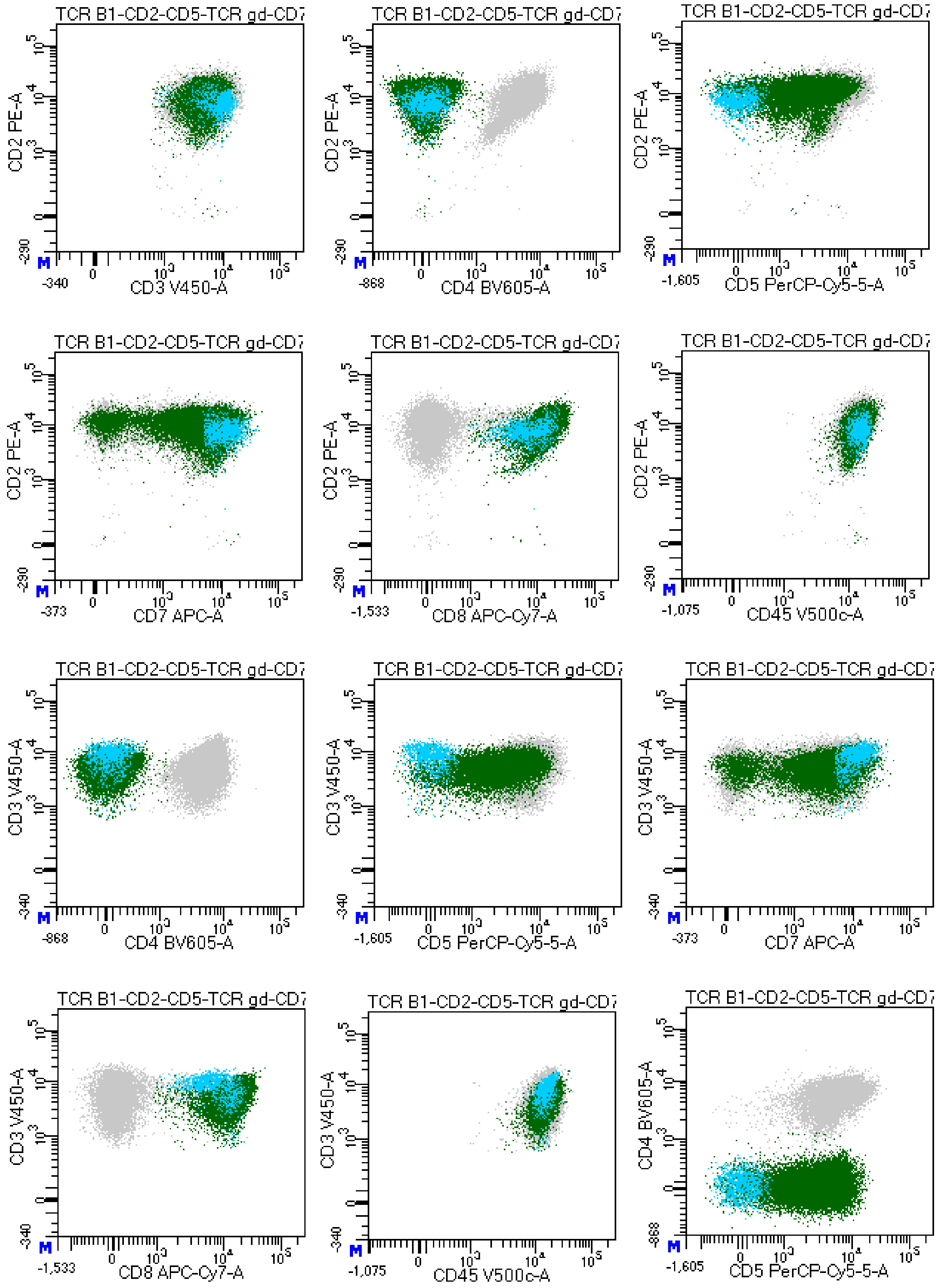

Initial manual analysis where only one CD8+/CD7+/CD5- T-CUS population (light blue) was found. (page 3/4)

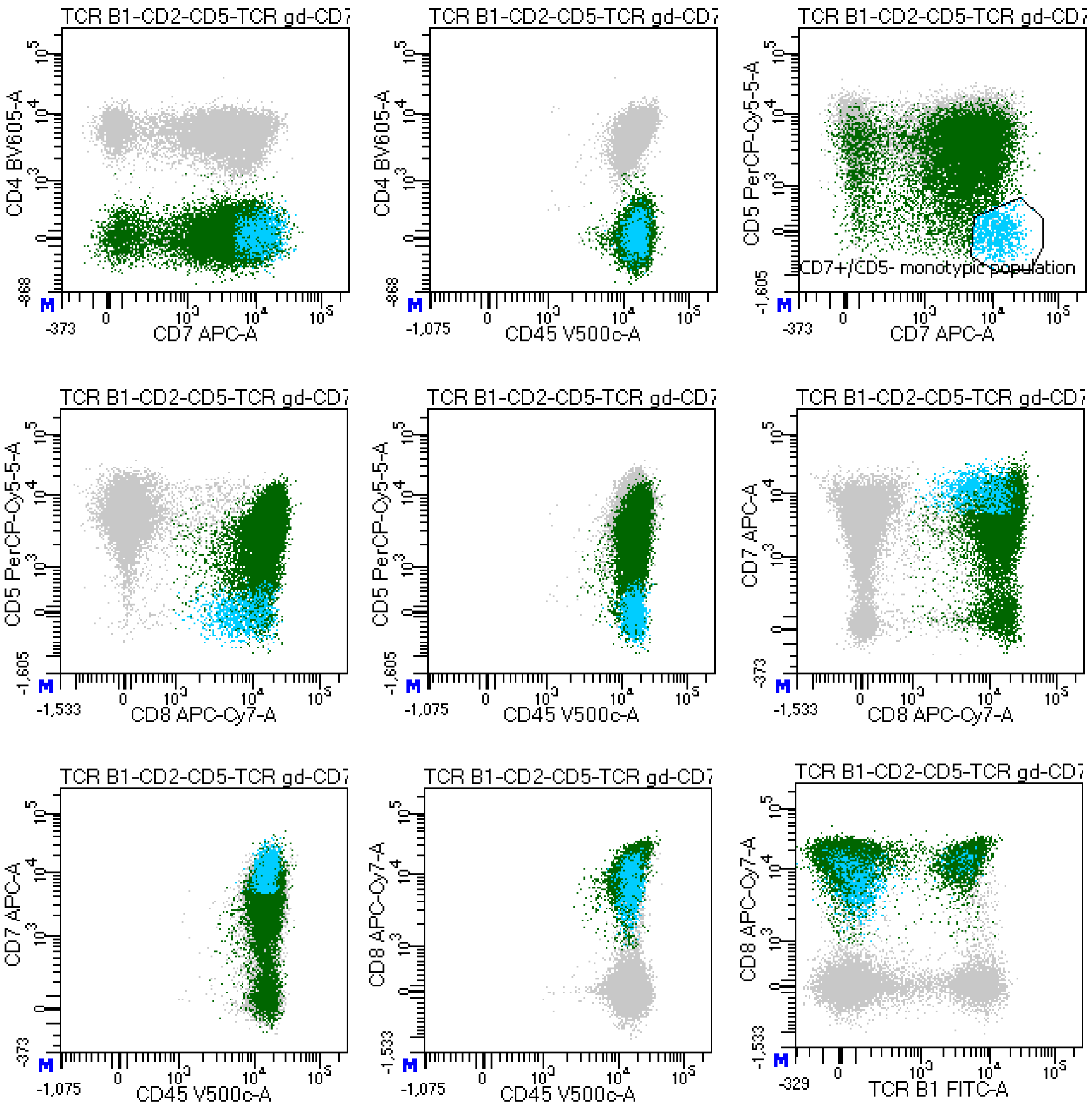

Initial manual analysis where only one CD8+/CD7+/CD5- T-CUS population (light blue) was found. **(page 4/4)**

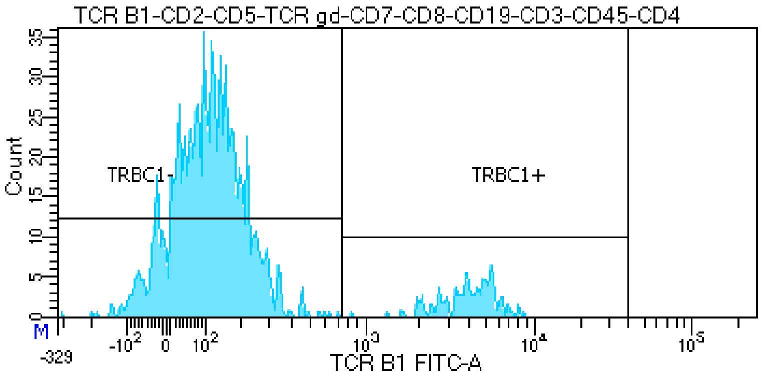

Tube: TCR B1-CD2-CD5-TCR gd-CD7-CD8-CD19-CD3-CD45-CD4

Population	#Events	%Parent	%Total
All Events	481,891	####	100.0
FSC/SSC	353,127	73.3	73.3
Single cells	344,065	97.4	71.4
CD45	331,980	96.5	68.9
CD3+	30,896	9.3	6.4
CD3+/TCRgd-	30,535	98.8	6.3
CD8+ cells	16,397	53.7	3.4
CD8+/CD7+/CD5- monotypic population	981	6.0	0.2
TRBC1-	878	89.5	0.2
TRBC1+	103	10.5	0.0

Revised manual analysis with lymphoma population (red). **(page 1/8)**

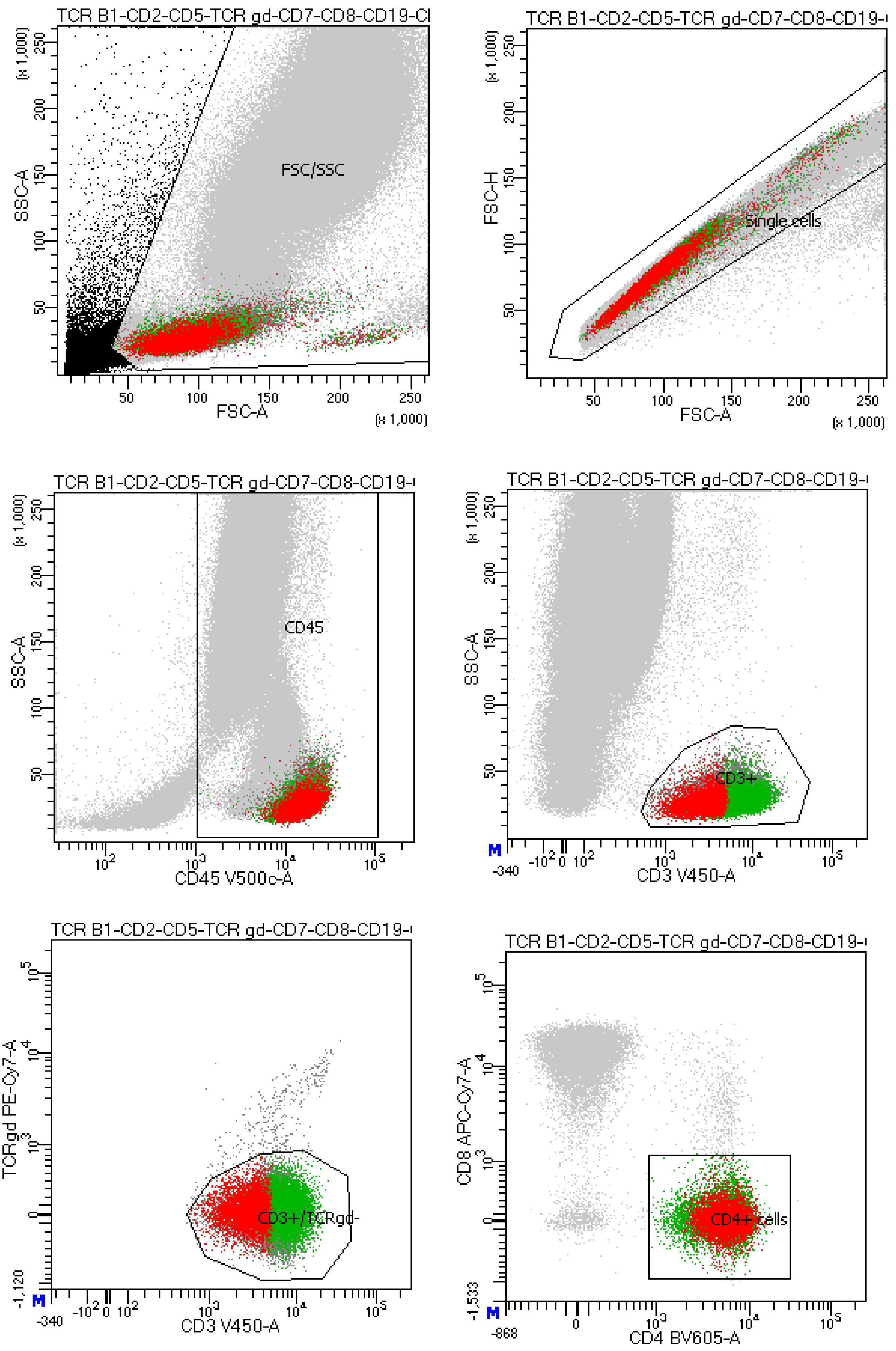

Revised manual analysis with lymphoma population (red).

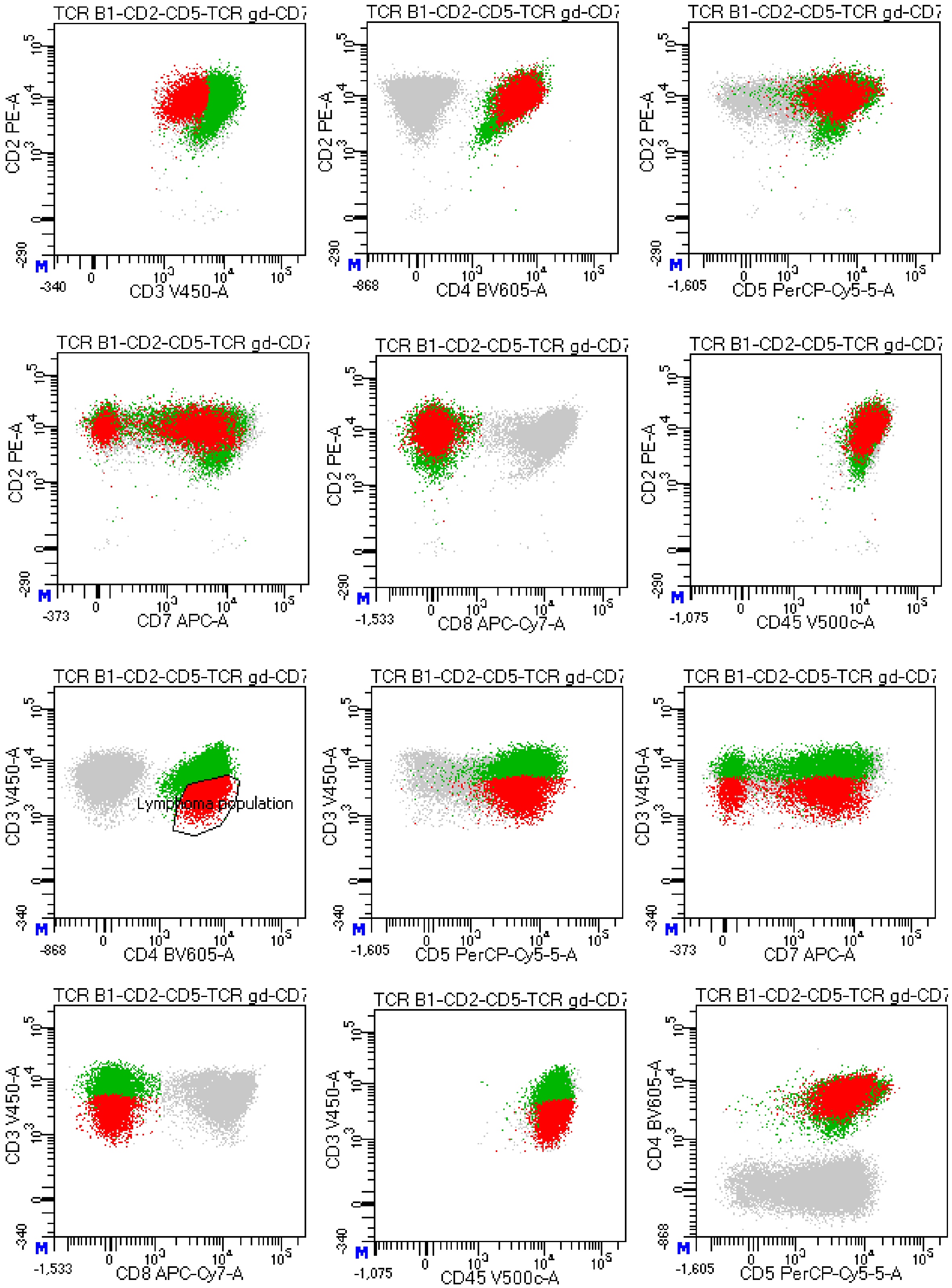

Revised manual analysis with lymphoma population (red). **(page 3/8)**

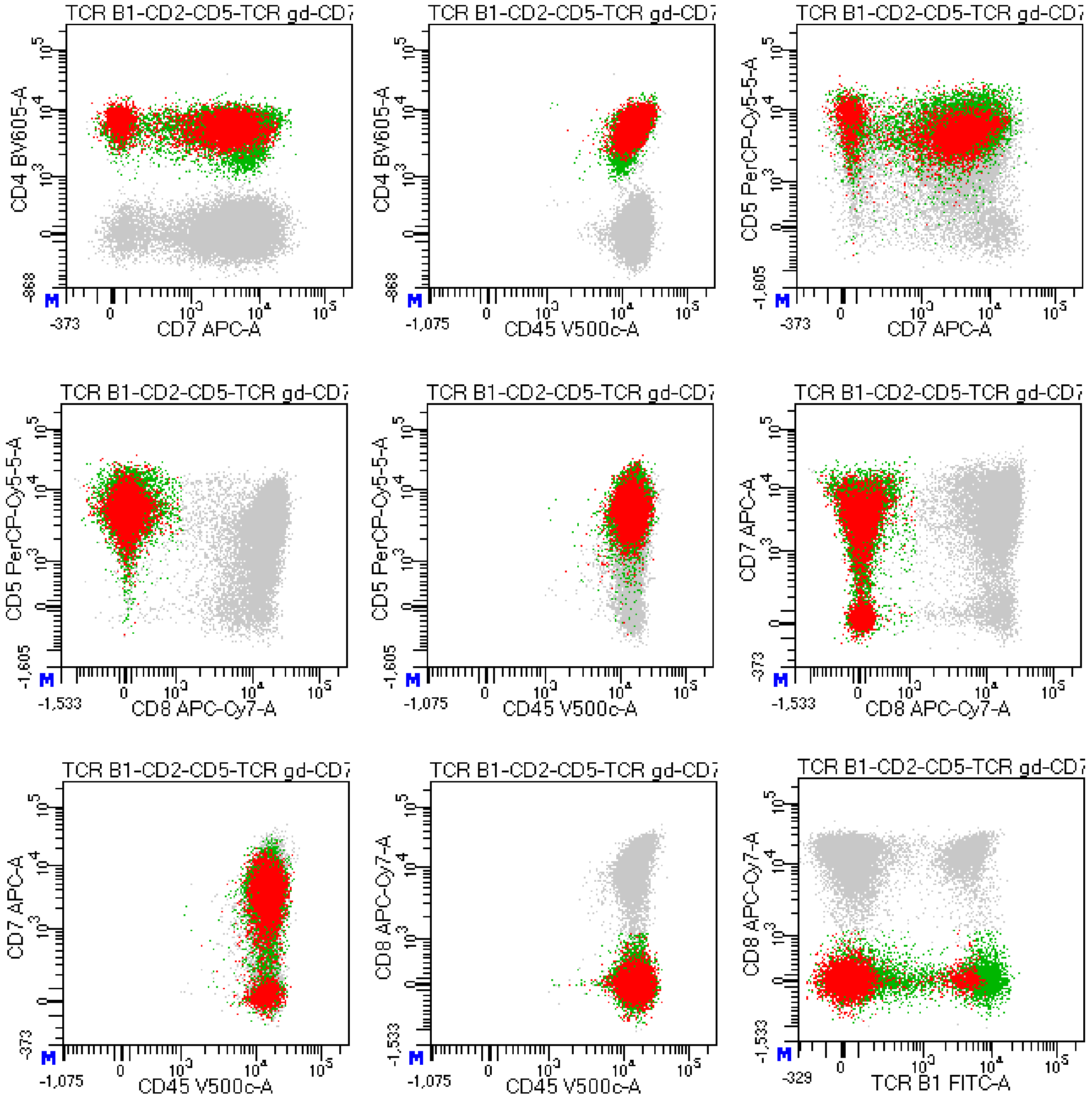

Revised manual analysis with lymphoma population (red). **(page 4/8)**

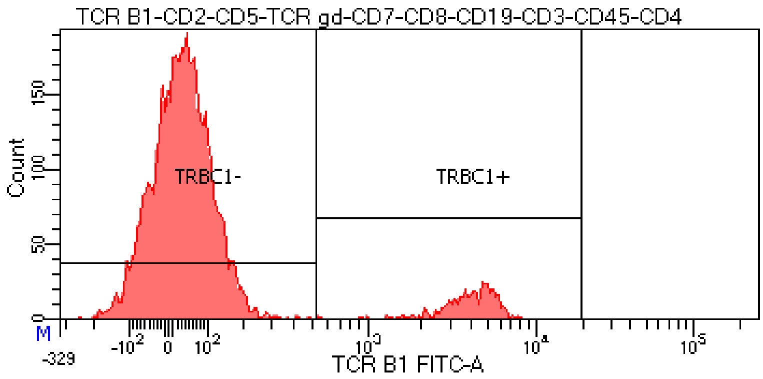

Tube: TCR B1-CD2-CD5-TCR gd-CD7-CD8-CD19-CD3-CD45-CD4

Population	#Events	%Parent	%Total
All Events	481,891	####	100.0
FSC/SSC	353,128	73.3	73.3
Single cells	344,066	97.4	71.4
CD45	331,980	96.5	68.9
CD3+	30,896	9.3	6.4
CD3+/TCRgd-	30,535	98.8	6.3
CD4+ cells	12,882	42.2	2.7
Lymphoma population	5,230	40.6	1.1
TRBC1-	4,727	90.4	1.0
TRBC1+	503	9.6	0.1

BD FACSDiva 9.1

Revised manual analysis with second CD8+/CD7-/CD5+ T-CUS population (dark blue).
(page 5/8)

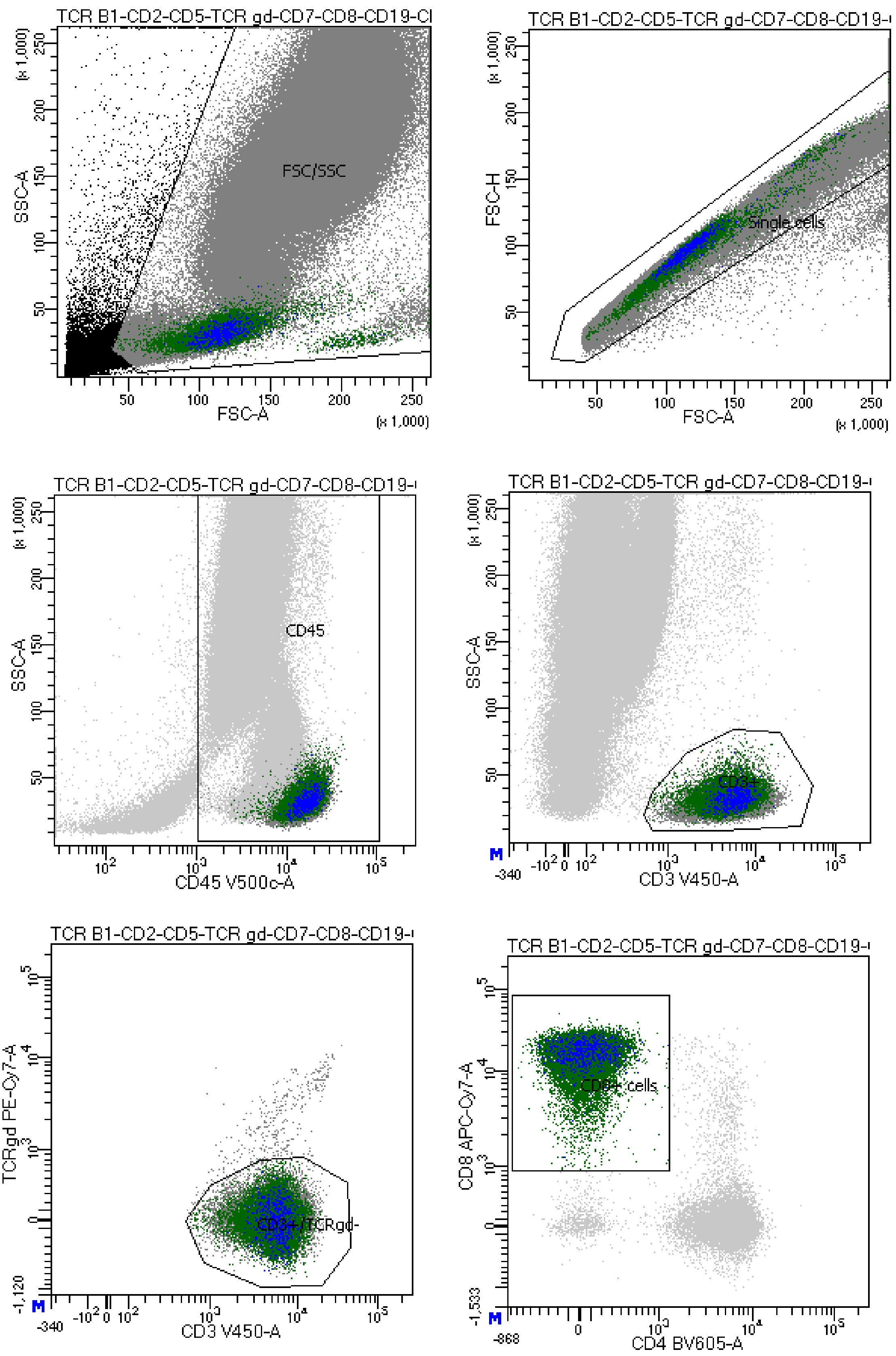

BD FACSDiva 9.1

Revised manual analysis with second CD8+/CD7-/CD5+ T-CUS population (dark blue). (page 6/8)

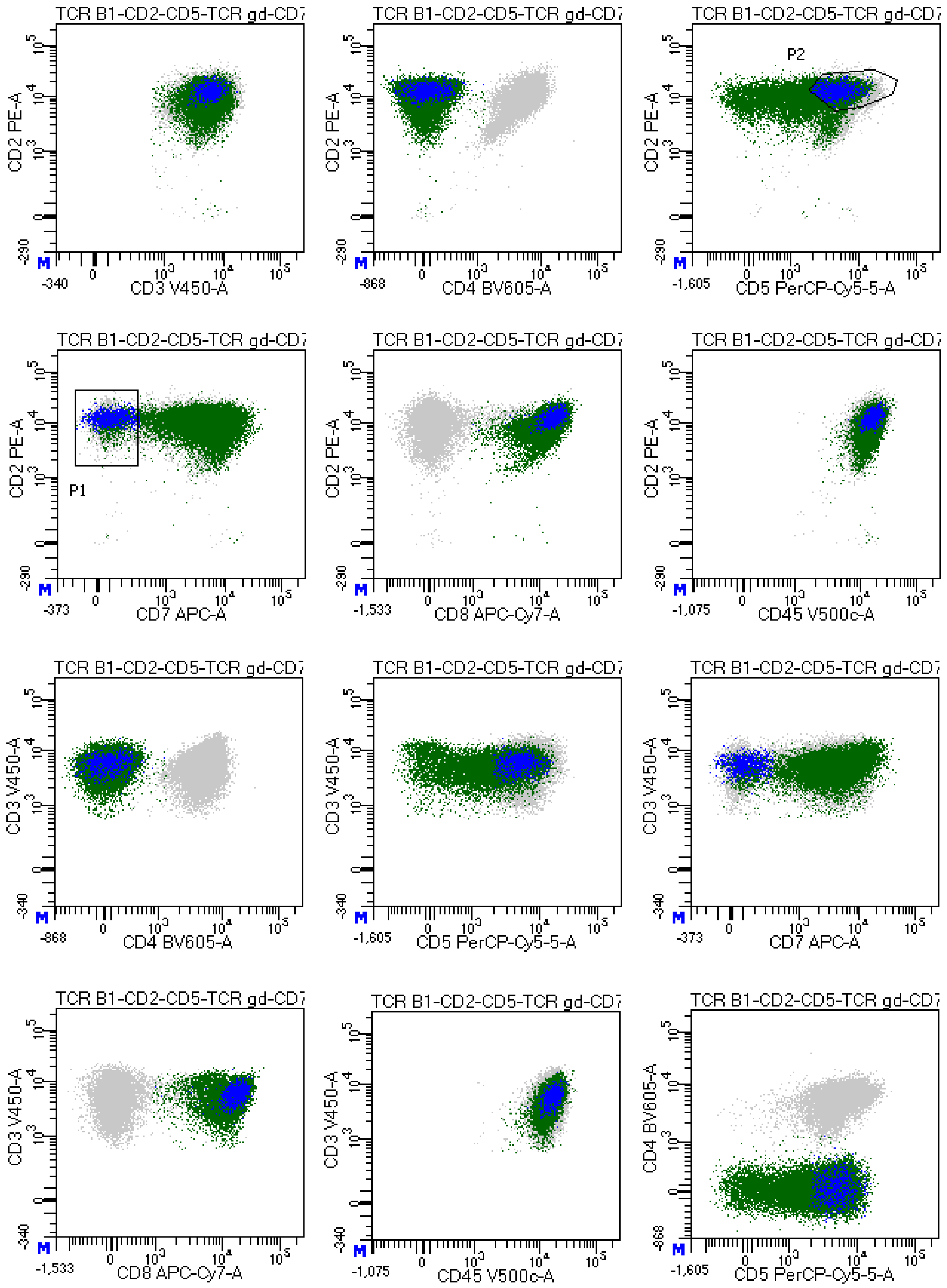

BD FACSDiva 9.1

Revised manual analysis with second CD8+/CD7-/CD5+ T-CUS population (dark blue). (page 7/8)

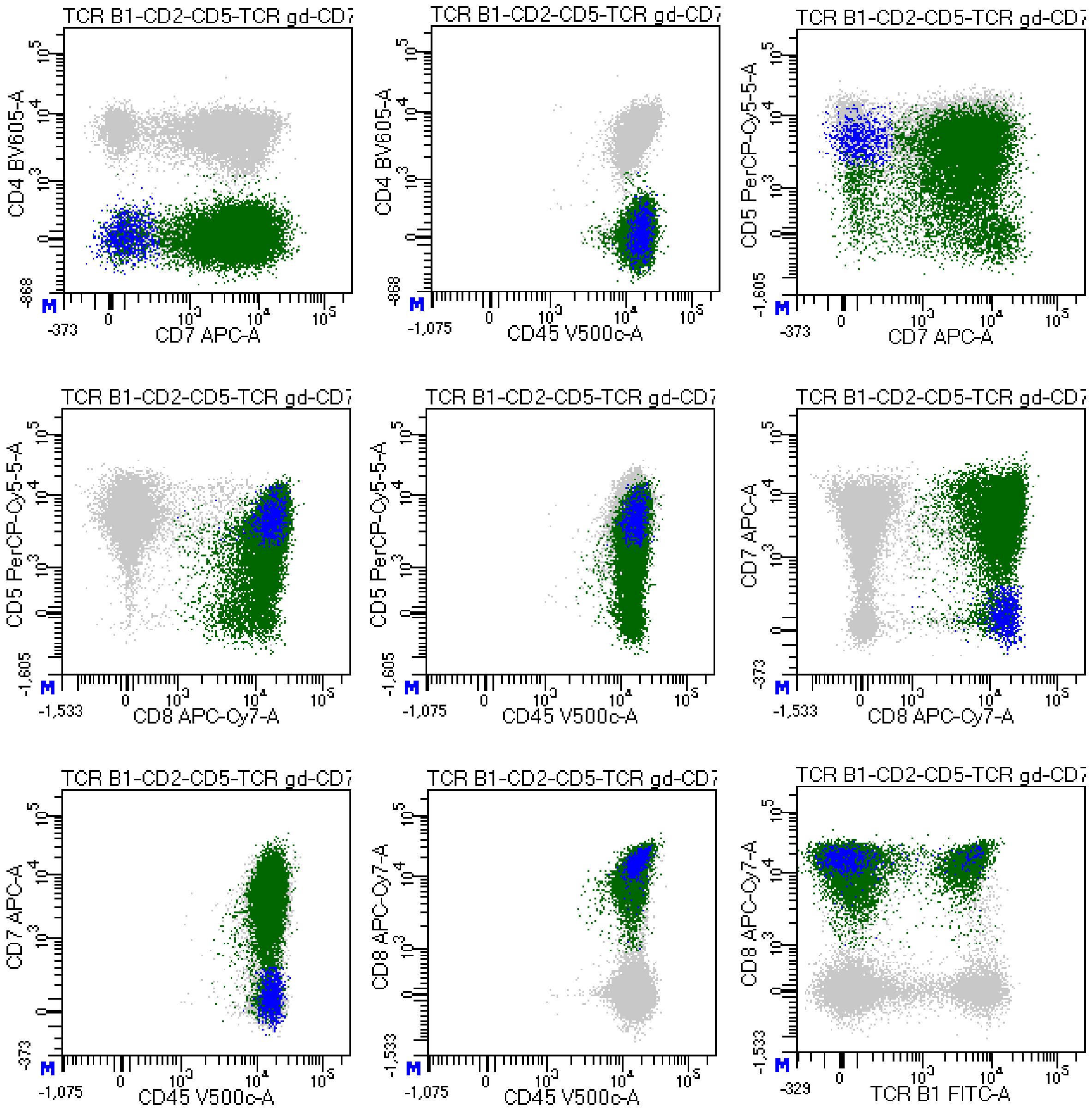

BD FACSDiva 9.1

Revised manual analysis with second CD8+/CD7-/CD5+ T-CUS population (dark blue).
(page 8/8)

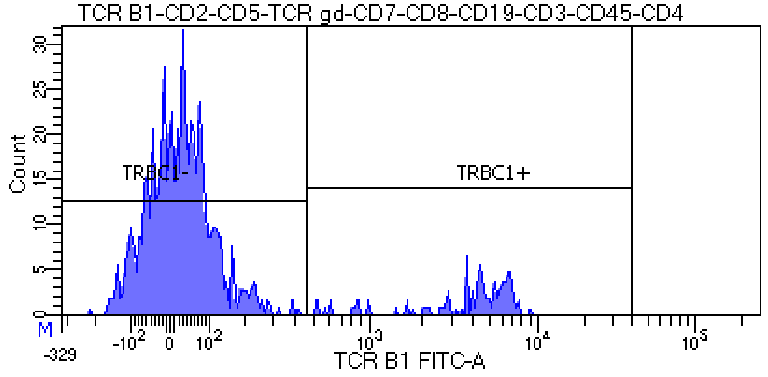

Tube: TCR B1-CD2-CD5-TCR gd-CD7-CD8-CD19-CD3-CD45-CD4

Population	#Events	%Parent	%Total
All Events	481,891	####	100.0
FSC/SSC	353,124	73.3	73.3
Single cells	344,062	97.4	71.4
CD45	331,980	96.5	68.9
CD3+	30,896	9.3	6.4
CD3+/TCRgd-	30,535	98.8	6.3
CD8+ cells	16,393	53.7	3.4
P1	1,454	8.9	0.3
P2	9,204	56.1	1.9
CD8+/CD7-/CD5+ monotypic population	699	4.3	0.1
TRBC1-	609	87.1	0.1
TRBC1+	90	12.9	0.0
